# Data Ethics Club: Creating a collaborative space to discuss data ethics

**DOI:** 10.1016/j.patter.2022.100537

**Published:** 2022-06-24

**Authors:** Nina H. Di Cara, Natalie Zelenka, Huw Day, Euan D.S. Bennet, Vanessa Hanschke, Valerio Maggio, Ola Michalec, Charles Radclyffe, Roman Shkunov, Emma Tonkin, Zoë Turner, Kamilla Wells

**Affiliations:** 1 MRC Integrative Epidemiology Unit, University of Bristol, Bristol BS8 2BN, UK; 2 Jean Golding Institute for Data Intensive Research, University of Bristol, Bristol BS8 1UH, UK; 3 School of Mathematics, University of Bristol, Bristol BS8 1UG, UK; 4 Bristol Veterinary School, University of Bristol, Bristol BS40 5DU, UK; 5 Department of Computer Science, University of Bristol, Bristol BS8 1UB, UK; 6 Bristol Cyber Security Group, University of Bristol, Bristol BS8 1UB, UK; 7 EthicsGrade Limited, 7-12 Tavistock Square, London WC1H 9BQ, UK; 8 Digital Health, University of Bristol, Bristol BS8 1UB, UK; 9 Nottinghamshire Healthcare NHS Foundation Trust, Nottingham NG3 6AA, UK; 10 Australian Public Service, Brisbane, QLD, Australia

**Keywords:** data ethics, responsible innovation, journal club, open science, open source, interdisciplinary

## Abstract

Awareness and management of ethical issues in data science are becoming crucial skills for data scientists. Discussion of contemporary issues in collaborative and interdisciplinary spaces is an engaging way to allow data-science work to be influenced by those with expertise in sociological fields and so improve the ability of data scientists to think critically about the ethics of their work. However, opportunities to do so are limited. Data Ethics Club is a fortnightly discussion group about data science and ethics whose community-generated resources are hosted publicly online. These include a collaborative list of materials around topics of interest and guides for leading an online data-ethics discussion group. Our meetings and resources are designed to reduce the barriers to learning, reflection, and critique on data science and ethics, with the broader aim of building ethics into the cultural fabric of quality data-science work.

## Highlights

• Data Ethics Club meetings encourage interdisciplinary discussion about data ethics
• Organizing openly means resources and ideas can be generated collaboratively
• Materials for organizing your own Data Ethics Club are provided
• Interested contributors are warmly invited to participate and make new suggestions

## The bigger picture

The Data Ethics Club aims to help data scientists, analysts, and other interested parties up skill in their knowledge of data ethics through discussions in an interdisciplinary setting that encourages individuals to explore their own ethical challenges and boundaries. Our aim is to make conversations about data ethics more common in data-science spaces and for these conversations to be informed by the critical work taking place in sociological and philosophical disciplines. Our longer-term ambitions are for our community to continue to grow and develop the resources we have created and for more groups in other locations and time zones to be established. By bringing data ethics to people in a range of roles and organizations, we can increase the awareness of ethics in our day-to-day settings and work toward preventing future injustices being aided and abetted by data science.

## Teaser

Safe and responsible data-science work should be influenced by knowledge from sociological fields, but there are limited opportunities for regular interdisciplinary conversation. Data Ethics Club (dataethicsclub.com) is a fortnightly discussion group about data science and ethics whose community-generated resources are hosted publicly online. The group’s meetings and resources reduce the barriers to learning, reflection, and critique on data science and ethics, with the broader aim of building ethics into the cultural fabric of quality data-science work.

## Introduction

Data science and digital technologies have a huge role in all of our day-to-day lives, and this role increases year after year. There are countless examples of how data science shapes and influences our lives: algorithms are used to decide who should have access to scarce public resources,^1^ mathematical models are being used in policing by attempting to “predict” crime before it happens,^2^ and a new labor market is emerging of those employed, and sometimes exploited, to produce data for the development of new digital tools.^3^ While many technologies are incredibly useful and represent exciting advances, the extent of their reach in our lives leaves those developing them with a huge amount of collective power over the way the world is modeled and interpreted using data.^4^

Those working in the ethics of emerging technologies have long discussed and been aware of this influence,^5^ but data scientists have not always recognized that the choices they make in their work, such as selecting projects or methods of modeling and analyzing data, as ethical decisions.^6^ We see three main reasons for this lack of engagement with data ethics on the part of data scientists. First, data-science culture tends to take a positivist stance that does not consider social lenses on technical problems as being important or worthwhile,^7^ which reduces considerations of ethics to compliance exercises rather than embedding them within data-science practice.^7^, ^8^, ^9^, ^10^, ^11^ Second, as a result, data-science education does not generally prepare data scientists for critical engagement with ethical literature and issues,^9^^,^^12^ leaving data scientists ill-equipped to understand, let alone apply, the knowledge produced in the social sciences.^9^ Third, there are rarely reciprocal opportunities for experts in data ethics from philosophy or sociology to influence the day-to-day practice of data scientists, even though this has been shown to be beneficial for all involved.^13^

In this descriptor article, we describe our approach addressing concerns about limited engagement with ethics by data scientists in the form of Data Ethics Club (https://dataethicsclub.com), a fortnightly discussion group with an accompanying open-source repository. Our overarching aim is to provide a replicable and structured opportunity for data scientists to engage with issues relating to data ethics and invite those working across multiple disciplines to discuss issues together. In doing so, we hope to promote responsibility, justice, and equity in applied data-science practice and to help build ethics into the cultural fabric of quality data-science work.

### What is Data Ethics Club?

**Figure 1:**
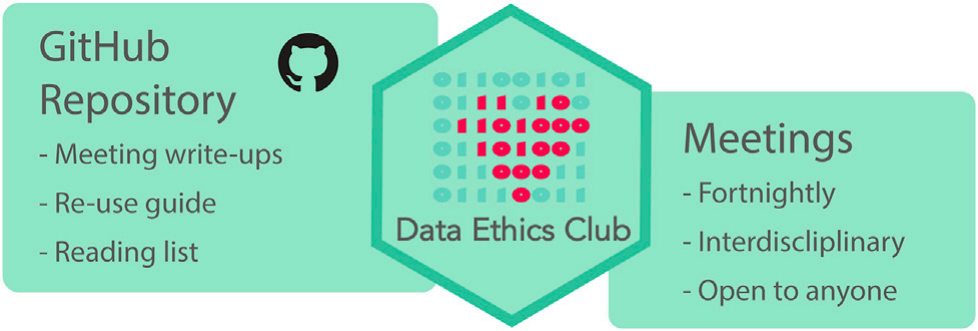
A visual overview of Data Ethics Club

Data Ethics Club is a fortnightly reading and discussion group held virtually that is currently hosted by University of Bristol staff and students (Figure 1). The hour-long lunchtime meeting is free to attend and open to everyone.

Data Ethics Club is organized around a central open-source repository (https://github.com/very-good-science/data-ethics-club) that has been developed collaboratively over time; this repository is the primary subject of this article. It includes our organizational materials, write-ups of our discussions, and our growing reading list. The repository encourages collaboration from new contributors who would like to make changes or additions to the repository or to re-use its contents. Our meetings are open to anyone who would like to join and regularly includes business owners, students, academics, industry-based data scientists, philosophers, policy makers, publishers, and more.

Specifically, Data Ethics Club meetings aim to do the following.

• Provide structured time and space for engaging with material about data ethics.
• Provide a collaborative environment where attendees can ask critical questions about the material and engage in discussions so that each of us can challenge our own assumptions.
• Bring together a varied group of people from across the data-science community and beyond.

### Motivation

Our incentive for creating Data Ethics Club was a desire for a space to discuss how we should practically *do* ethics as data scientists,^10^ with a broad view of what ethical practice means, from engaging with different types of knowledge beyond those where we are comfortable^7^^,^^14^ to how we approach structural issues like racial inequality in our work.^15^ Other similar ideas for reading groups and reading lists about data ethics have been created previously by groups such as DataKind’s Data Ethics Book Club in A Box^16^ or the Data Feminism Reading Group.^17^ However, we found that most existing opportunities were restricted to those from specific organizations or only operated for a short period of time. Peiffer and colleagues^8^ developed a model for discussing ethics 15 minutes a week after a research-group meeting and found this helped group members to consider ethics regularly in their science practice, more so than one-off training or a short course. We similarly wanted a regular and ongoing opportunity for discussion that was akin to an academic journal club^18^^,^^19^ but that, unlike a typical academic lab group or department, explicitly invited a wide range of attendees from a range of disciplinary and professional backgrounds. Building interdisciplinary spaces where people from different areas can come together is a crucial part of how we can begin to encourage data scientists to engage with critical material from fields^13^^,^^20^ and is a vital component of ethics education in data science.^7^^,^^9^ Having these spaces, which may be referred to as “trading zones”^21^ or “communities of practice,”^22^ allows important lessons and concepts learned in the field of ethics to reach those who are working in applied settings, which was what we sought to achieve.

The group setting of Data Ethics Club, and the emphasis on discussions rather than lectures, is an acknowledgement that ethics is fundamentally co-constructed and relative;^20^ one person with one perspective simply cannot have all the answers.^23^^,^^24^ As such, participation in a discussion group becomes an awareness-raising exercise that allows attendees to feel out the boundaries of what is acceptable to who and why. Most importantly, we can start to understand what is important to ourselves, what influence that may have on our own work, and how this may affect others.^24^ This does require a willingness on the part of data scientists to engage with critical questions about ethics of our work. Some data scientists do this already,^6^ though there is a spectrum of ethical considerations that may go from nothing at all, to treating bias as a technical issue, to fully engaging with data ethics as a professional practice. By creating a structured environment for these questions and considerations, we hoped to make it easier for data scientists to integrate ethical thinking into their professional practice.

## Results

The Data Ethics Club repository is hosted on GitHub^2^ and has been in ongoing development since October 2020, with collaboration from approximately 35 different people across academia, government services, and private industry. Their contributions have been toward the three primary outputs of the repository.

• A repository structure, which can be copied for anyone to start their own group, that contains organizational materials and an integrated website.
• Write-ups from meetings of the Data Ethics Club that are co-produced by the attendees.
• A reading list of discussion materials around the theme of data ethics.

These three outputs can all be found in the “site” sub-folder of the repository and are organized into folders that are directly reflected in the website’s structure. The project remains live and continues to develop and grow as time goes on; however, a static release with a Digital Object Identifier made at the time of writing is available (https://doi.org/10.5281/zenodo.6243803). The repository is licensed with a CC-BY-3.0 license,^25^ which allows people to re-use and re-mix the content with accreditation, in line with our aims. We describe the main outputs of the repository in more detail below.

### Online journal club repository materials

The first output of the Data Ethics Club is the guides and materials for starting and running another group, specifically as an online journal club. We have created a “How To” sub-section of the repository that contains all of our resources ready for re-use. This includes a template for a Code of Conduct, instructions on necessary tasks before the meeting, the structure and timing of the meeting itself, and the software we use to help facilitate the group. These guides also ensure that the organization of the group is transparent and help to on board and support new organizers.

The repository is hosted on GitHub and integrated with several useful features that could be re-used by forking or downloading the materials for those who do not use GitHub. Firstly, there is a custom issue template, designed for suggesting new reading materials, that invites the suggester to contribute three questions to be used as discussion prompts. Secondly, the repository has integrated the All Contributors specification,^26^ which is a tool that allows us to record everyone who contributes to the repository, even if their contribution is not something that GitHub would typically recognize. We wanted to recognize the value of attending meetings, emailing us suggestions, making artwork for the repository, and generating ideas. To illustrate, only 11 of our 35 current contributors would be automatically recognized by GitHub by having made commits to the repository otherwise. Lastly the contents of the repository also generate a website (https://dataethicsclub.com) that is served directly by GitHub pages. The website is rendered by Sphinx^27^ and is re-built and deployed every time a new commit is added to the repository using Github Actions. This makes it very simple to keep the website up to date, even for those who are unfamiliar with front-end web development, since all of the website documentation is based on MyST flavored markdown.^28^ By hosting the materials openly on GitHub, we appeal to data scientists and enable them to contribute easily; by mirroring these materials on a public-facing website, they become more accessible to those who are not familiar with GitHub.

### Data Ethics Club write-ups

At each meeting, we encourage attendees to write down salient points from their discussions in an open online HackMD document that allows us all to collaborate on the same document in real time. (HackMD is a collaborative web-based markdown document editor [https://hackmd.io/]) These notes are then written up by one of the organizers and compiled into an online article that summarizes the themes of our discussions and the multiplicity of perspectives that these inspire, as well as suggestions from attendees for further reading; there are currently 22 online. The write-ups differ from a traditional opinion piece in that often they propose more questions than answers, acknowledge the different ideas and viewpoints from various members of the group, and aim to provoke thought just as much as they seek to inform. This is useful for members hoping to get more of an insight into the discussion from other breakout rooms or for those who missed the discussion and are wanting to catch up. We also use these write-ups to share our thoughts and feedback with those who wrote or produced the content we discuss. For example, after our discussion on The Rise of Private Spies (https://dataethicsclub.com/contents/write_ups/2021/28-07-21_writeup.html), we received positive feedback from an employee from the organization Bellingcat, which was featured heavily in the discussion.

### Reading list

The reading list for Data Ethics Club has been put together collaboratively by those with an interest in the topic who wanted to contribute to the initiative. Since the list is open source, it is intended to expand in the future with the addition of new resources and sub-sections whenever they are suggested. We aim to understand not just the philosophy of data ethics but also where our established norms came from in data science as well as current examples of implementations of data ethics. We also consider research culture to be a crucial element of ethical data science and aim to include content that requires us to consider structural inequalities in our individual settings.

The sub-sections that the materials are sorted into, described in our current reading list sub-sections, described in Table 1, were developed organically by N.H.D.C. and N.Z. over the period of a year. This was a pragmatic step to organize the volume of content into manageable, thematically similar groups, and so the sections do not necessarily cover the entirety of the field of data ethics in their current form. Additionally, as with all processes of categorization, certain standpoints and values are implicitly embedded in what is explicitly named and what is not.^29^ In creating the sections, we were led by our technical perspectives and considered the different levels of entry that interested readers might have, what topics might be of interest to applied data scientists, and also the separation of types of material. For instance, organizational reports and highly technical pieces have distinct sections. Alternative approaches to categorization could yield promising directions that reflect a greater focus on ethical impacts and name harms more explicitly, especially those to marginalized communities. As mentioned later in future developments, we plan to develop our website to allow for each piece to be allocated multiple categories, which would enable these multiple perspectives on categorization to be implemented.

**Table 1:** A list of the subsections of the reading list, with descriptions of the content included in each

Section	Description
What is data ethics?	Pieces that map the boundaries of data ethics and introduce the concept at a broad level
The nature of data	Exploring how we understand the data we use as a tool embedded with assumptions rather than a static, objective object
Moral philosophy for data science	Moral philosophy applied to data-science questions and ethical dilemmas
History of data science	The origins of data science and consideration of inherited norms and principles that we may take for granted
Algorithmic decision making	Discussing practical applications of algorithmic decisions, with its uses, misuses, and limitations
Environmental costs and considerations	Considerations of how computing can damage our environment and ways we can try to reduce this impact
Privacy and surveillance	The implications of technology for our privacy and discussing where we should find ethical boundaries in addition to existing legal guidelines
Data ethics in the public and private sectors	Many organizations have produced reports or ethical guidelines for their particular contexts, which are collected here
Research culture	Pieces related to the settings we research in, including pieces on team science, open science, white supremacy, and colonialism
Ethics in action	Examples of places where data ethics has been “played out” in the real world or theories and frameworks on how to practically implement good ethical practices
Field specific	Sub-sections around natural language processing, computer vision, and explainable artificial intelligence (AI)/machine learning (ML), which contain pieces specific to these fields; these tend to be more technically focused

The content included in the reading list is intended to be relatively accessible to those who are approaching the topic for the first time. It varies in format, including journal articles, government reports, book chapters, YouTube videos, and podcasts. Audio, video, long-form (like book chapters), very short-form (like poems), or closed-access content are indicated in the list using an emoji key to help people find what they need.

## Discussion

At the time of writing, Data Ethics Club has been running for over a year, and in this time, we have had been having regular fortnightly meetings and continually adding to the repository. We have benefited from a wide range of people being part of our community, from those who work in different sectors, to those living in different countries (from the UK where the organizers are based to Peru, Australia, the United States, India, and Italy), to experts in data ethics, deep learning, and software engineering coming together. The Data Ethics Club repository is shared with a CC-BY licence to ensure that others can re-use it and benefit from the collaborative effort that has gone into creating it. We also hope others will be inspired to contribute their own ideas to the repository and help us to improve it (see Box 1). In the following sections, we briefly discuss some of what we have learned in setting up and leading this project with the aim for our reflections to be useful to others, as well as outlining some of the current limitations of the project and planned future developments.

Box 1 **How to contribute to and use the Data Ethics Club repository**

Data Ethics Club is a collaborative online project that warmly invites new people to join the community and use its resources. Ways you can get involved include the following.

• Suggest new content for the reading list (via e-mail or GitHub).
• Attend, lead, or write up a meeting.
• Create or review pull requests in order to, for example, update the website for the next meeting, put more suggestions on the reading list, or ensure all contributors are credited.
• Use the Data Ethics Club resources to run your own discussion group or to create a reading list of your own or for students.
• Tell us how you used the resources, or give us feedback to improve on them.

### Impact

Data Ethics Club aims to have a positive impact both on those who attend and are part of our community and externally by being part of change in wider data-science culture. Feedback from meeting attendees, gathered through a small survey as well as unsolicited emails and comments, has been extremely positive. Attendees particularly highlighted the value of having the opportunity to discuss data ethics with a diverse group of people. The type of diversity in question varies, from a range of subject specialisms, to workplace contexts, to the countries our attendees live in. As mentioned previously, we believe the real benefit in discussing ethics in a group environment is having the opportunity to see outside of our own perspectives, and hosting a variety of attendees is an important factor in being able to achieve this. However, there is always work to do in this area, and we outline the impacts of attendee self-selection bias in the limitations section below.

The Data Ethics Club format has also given us a voice as a community of people with interests and expertise in this area, which we can use to affect change. For instance, sending feedback based on our discussion writeups to calls such as those from the UK Statistics Authority about their proposed data-ethics landscape review (https://dataethicsclub.com/contents/write_ups/2021/14-04-21_writeup.html) and receiving feedback from organizations we have discussed such as Bellingcat (https://dataethicsclub.com/contents/write_ups/2021/28-07-21_writeup.html) that they found our written reflections useful. Having a community also gives many of us more confidence to push back against decisions that we believe may be harmful, have difficult conversations with colleagues, or to go against the status quo. Another result of the Data Ethics Club community has been the development of the Data Hazards project, which aims to help data scientists have effective interdisciplinary discussions about ethics at all stages of their work (https://datahazards.com/).

Lastly, as our online collection of resources grows, they have begun to be re-used. An aligned discussion group aimed at undergraduates has been started by one of our attendees, who is an undergraduate themselves. The format for our reading list has also been used by a Data and Society reading group formed by and for UK civil servants, hosted by the Government Digital Service (https://github.com/alphagov/data-ethics-and-society-reading-group/blob/main/READING-LIST.md).

### Collaborative ethos

Following the example of other open-source projects that we admire (for instance, see The Turing Way^30^), the repository and the group meetings of Data Ethics Club try to take an ethos of collaboration and equity. This means that, as much as possible, we want to share opportunities to own and lead the project, give a variety of options for how to get involved, and give thanks and credit for everyone’s contributions. To do this, we have made intentional choices about how to work with others on this project, such as the following.

• Making the reading list (and everything else) open source so that others can use it.
• Using the All Contributors specification to credit everybody, including those not on GitHub, so that all labor associated with the project is recognized.
• Giving people the option to contribute by writing as well as speaking to make getting involved as accessible as possible.
• Making it easy for people to suggest papers, or get involved in organizing, by sharing contact information regularly and inviting contributions often through different routes (e.g., e-mail, GitHub, Twitter).
• Providing different ways for people to get involved that require various levels of commitment so that not only those with lots of free time to give are represented in the repository’s development.
• Provide opportunities for people to give feedback on how the group is run and be open to making changes.

In doing so, we aim to practice the values that we want to encourage in our own work and in data-science practice more generally and to emphasize that opportunities for collaboration and sharing power can be found in how we organize, as well as in our technical work.

### Tips for coordinating an online journal club

In organizing Data Ethics Club, we have developed our processes for organizing and running an online discussion group. The Data Ethics Club was established during the coronavirus pandemic, and so online was the default option at the time; however, we have found this has actually had a huge benefit. It has made it easy to reach beyond our immediate institution and even outside of the country; we tend to have people from at least three different countries at each meeting. Based on this, the following is some advice for organizing a group online based on our experiences.

• Make use of timezone sensitive tools. We use Time and Date (https://timeanddate.com/) to share the time of our next meeting, which makes it easy for people in different time zones to work out if they can come along.
• Make use of breakout rooms. We have found it works well to host several small group (4 to 6 people) discussions for the majority of the meeting and then use the larger group setting to hear from each group and gather general feedback. This enables everyone to participate fully in the conversation while also hearing a wide range of views. This is evident in the wildly different paths that conversation takes in each breakout room.
• Establish and share the Code of Conduct with a reminder at each session. Other groups are welcome to re-use the one we have shared on our repository that was adapted from other journal clubs and those from open-source communities.
• Establish and share an accessibility plan with a reminder at each session and in contribution guides.
• Make sure that the discussion content is not too long or complex. People do their best to read or view it before attending, but not everyone will have, and so something that does not present a barrier to getting involved and that can be quickly summarized at the beginning is advisable.
• Send regular reminders. We especially find that reminders early on the day of the meeting are useful.
• Enable people to provide anonymous feedback on the discussion and rate the discussion material. We use polls in Zoom for this purpose.

### Limitations

While we aim for our materials and repository to be as useful, representative, and inclusive as possible, there are limitations to the repository that we intend to continue working on as the project matures and develops.

First, to edit or use the repository, a certain level of familiarity with *git* and GitHub is needed to be able to submit an issue, a pull request, or to reproduce and edit the repository for one’s own purposes. We encourage people to get in touch via e-mail for support and also provide detailed contribution guidelines to reduce this barrier, but it may nonetheless prevent input from those with useful insights who are less familiar with the tools that we use to organize.

Second, the repository and group are UK centric, English speaking, and holds meetings at GMT-friendly hours. Inevitably, this restricts the attendance of those from areas that do not share work hours in our time zone, who are not English speaking, or who do not have reliable high-speed-internet access. By making our materials open and well documented, we hope to enable and encourage Data Ethics Club groups to develop in other countries and time zones.

Third, by deciding what we discuss via vote, we are at risk of the tyranny of the majority whereby minority interests are unlikely to win the vote. We try to counter this by holding themed meetings, where all content choices relate to a particular topic or discipline.

Lastly, we are aware that those who to invest time in regularly attending and contributing to the Data Ethics Club community are likely to be those with an existing interest in improving data-science culture. As such, our community is likely to be a self-selecting sample of much wider data-science and data-ethics communities. Similarly, the community is also most likely to be made up of those who have the time, space, and access to take part.

### Future developments

Our planned future developments fall into two categories: additional formats and development of outputs.

We have plans to expand the trialed format of Data Ethics Club events to include, for example, Data Ethics “Clinics,” where attendees can bring their own data-ethics dilemmas (perhaps using the Data Hazards materials), a Data Ethics Club Unconference, and public outreach to involve citizens in our conversations.

We expect the existing website outputs to continue to grow and broaden over time via our regular meetings and calls for new materials. In addition, we continue to extend invites to new groups of attendees to enrich the resources and quality of the discussion. We also plan to trial new processes such as fairer voting systems and website resources such as a search function for the reading list that allows content to belong to multiple categories.

### Conclusions

The Data Ethics Club repository, and its associated community, will continue to develop as time goes on. For data ethics to become embedded in our daily practices, we need to understand that each of us has a role in preventing harm from data-science outputs and realize that data ethics is a practice for all of us to engage in collaboratively. Our impacts so far illustrate that there is a valuable role for a group like Data Ethics Club to support this practice. We hope that this repository and the Data Ethics Club will be a starting point for sharing data ethics with other data scientists and giving those with expertize in data ethics a chance to share their knowledge with the people who put it into action.

## Experimental procedures

### Resource availability

#### Lead contact

Requests for further information should be directed to Nina H. Di Cara (ninadicara@protonmail.com).

#### Materials availability

No materials were used in this study.

## Data Availability

The Data Ethics Club repository is an open-source project, and so the latest version will always be publicly available at https://github.com/very-good-science/data-ethics-club. The contents of the repository are served as a website at https://dataethicsclub.com/. The DOI for the version of the repository referred to in this article is available at https://doi.org/10.5281/zenodo.6243803.

## References

[1] Eubanks V. (2018).

[2] Tench S., Fry H., Gill P. (2016). Spatio-temporal patterns of IED usage by the provisional Irish republican army. Eur. J. Appl. Math..

[3] Stanley S. (2021). Living in the hidden realm of AI: the worker’s perspective. https://news.techworkerscoalition.org/2021/03/09/issue-5/.

[4] Costanza-Chock S. (2020).

[5] Moor J.H. (1985). What is computer ethics?. Metaphilosophy.

[6] Barocas S., Boyd D. (2017). Engaging the ethics of data science in practice. Commun. ACM.

[7] Raji I.D., Scheuerman M.K., Amironesei R. (2021). Proceedings of the 2021 ACM conference on fairness, accountability, and transparency.

[8] Peiffer A.M., Hugenschmidt C.E., Laurienti P.J. (2011). Ethics in 15 min per week. Sci. Eng. Ethics.

[9] Bates J., Cameron D., Checco A., Clough P., Hopfgartner F., Mazumdar S., Sbaffi L., Stordy P., Vega de León A. de la (2020). Proceedings of the 2020 conference on fairness, accountability, and transparency.

[10] Keller S.A., Shipp S.S., Schroeder A.D., Korkmaz G. (2020). Doing data science: a framework and case study. Harvard Data Science Review.

[11] Friedman B., Kahn P.H. (2007). The human-computer interaction handbook.

[12] Saltz J.S., Dewar N.I., Heckman R. (2018). Proceedings of the 49th ACM technical symposium on computer science education.

[13] McGregor J., Wetmore J.M. (2009). Researching and teaching the ethics and social implications of emerging technologies in the laboratory. Nanoethics.

[14] Lewis J.E., Abdilla A., Arista N., Baker K., Benesiinaabandan S., Brown M., Cheung M., Coleman M., Cordes A., Davison J. (2020). Indigenous protocol and artificial intelligence position paper. https://www.indigenous-ai.net/position-paper/.

[15] Daniels J., Nkonde M., Mir D. (2019). Advancing racial literacy in tech. https://datasociety.net/wp-content/uploads/2019/05/Racial_Literacy_Tech_Final_0522.pdf.

[16] Data Kind (2020). Data ethics book club in a box. https://github.com/DataKind-UK/data-ethics-book-club-in-a-box.

[17] D’ignazio C., Klein L.F. (2020).

[18] Linzer M. (1987). The journal club and medical education: over one hundred years of unrecorded history. Postgrad. Med..

[19] Orben A. (2019). A journal club to fix science. Nature.

[20] Wylie C.D. (2020). Who should do data ethics?. Patterns.

[21] Gorman M.E. (2010).

[22] Research in Practice (2021). Practice tool: developing a community of practice in your organization. https://practice-supervisors.rip.org.uk/wp-content/uploads/2021/01/StS_PT_Developing_a_community_of_practice_in_your_org_Final.pdf.

[23] Harding S. (2016).

[24] Birhane A. (2021). Algorithmic injustice: a relational ethics approach. Patterns.

[25] Attribution 3.0 international (CC BY 3.0) (2007). https://creativecommons.org/licenses/by/3.0/legalcode.

[26] All contributors (version 2.16). https://github.com/all-contributors/all-contributors.

[27] Brandl G. (2021). Sphinx documentation. http://sphinx-doc.org/sphinx.pdf.

[28] Community E.B. (2020). Jupyter book (zenodo).

[29] Bowker G.C., Star S.L. (2000).

[30] Turing Way Community (2022). The Turing Way: a handbook for reproducible data science. Zenodo.

